# The effect of antidepressants on glioblastoma survival: A systematic review and meta-analysis

**DOI:** 10.1093/noajnl/vdaf075

**Published:** 2025-06-14

**Authors:** Yifei Sun, Mohammad Hamo, Travis Atchley, Burt Nabors, James Markert, Dagoberto Estevez-Ordonez

**Affiliations:** Department of Neurosurgery, Marnix E. Heersink School of Medicine, University of Alabama at Birmingham, Birmingham, AL, USA; Department of Neurosurgery, Marnix E. Heersink School of Medicine, University of Alabama at Birmingham, Birmingham, AL, USA; Department of Neurosurgery, University of Alabama at Birmingham, Birmingham, AL, USA; Division of Neuro-Oncology, University of Alabama at Birmingham, Birmingham, AL, USA; Department of Neurology, O’Neal Comprehensive Cancer Center, University of Alabama at Birmingham, Birmingham, AL, USA; Department of Neurosurgery, University of Alabama at Birmingham, Birmingham, AL, USA; Department of Neurosurgery, University of Alabama at Birmingham, Birmingham, AL, USA

**Keywords:** antidepressants, depression, glioblastoma, SSRIs, survival

## Abstract

Symptoms of depression are highly prevalent among patients with glioblastoma and have been associated with poor outcomes. In addition to managing depression symptoms, preclinical and clinical studies suggest that antidepressants may inhibit glioblastoma progression However, the effect of antidepressants on survival is unknown and results from existing studies are conflicting. We conducted a systematic review and meta-analysis to investigate the effect of antidepressant therapy on glioblastoma survival. We queried PubMed, Embase, Scopus, PsycINFO, and Web of Science from inception to March 2024 for studies investigating the overall survival of patients with glioblastoma treated with antidepressants. Results were combined using a restricted maximum-likelihood estimation random effects model. We identified 6 observational studies meeting inclusion criteria with 8,269 patients. Of these, 1,093 (13%) were treated with antidepressant therapy at some point after their glioblastoma diagnosis. Antidepressant therapy was not significantly associated with overall survival (HR 1.27, 95% CI 0.90–1.79, *P* = .17). In a subanalysis of studies that specified whether selective serotonin reuptake inhibitors were utilized, SSRI usage specifically was also not associated with overall survival (HR 1.08, 95% CI 0.58–2.03, *P* = .81). We observed considerable heterogeneity (I^2^ = 92.7%, *P* < .001). Antidepressants were not significantly associated with overall survival in patients with glioblastoma. However, our meta-analysis was limited by significant heterogeneity observed across studies. The effect of antidepressants on outcomes in patients with glioblastoma remains uncertain. We observed conflicting results in the literature, with some studies suggesting decreased survival. Therefore, the role of antidepressants in patients with glioblastoma warrants further investigation.

**Key Points**

- Antidepressant therapy may be associated with improved outcomes
- Further evidence is needed to assess a survival benefit associated with antidepressant therapy

Glioblastoma is the most common primary malignant tumor of the brain, comprising nearly 50% of all central nervous system tumors. Despite improvements in care, it continues to carry a poor prognosis, with 5-year survival rates under 7% and an average survival time of 8 months postdiagnosis.^1^ Despite advancements in the treatment of glioblastoma, prognosis remains poor. Depression is a common comorbidity of patients with glioblastoma, with some studies suggesting rates ranging from 33% to 44%.^2^ Consequently, much of literature suggests that depression is associated with worsened outcomes in glioblastoma. However, management of these depression symptoms remains controversial, with little data available to conclusively support the treatment or nontreatment of depression symptoms in patients with glioblastoma. Some studies suggest increased survival benefit, while others show the opposite.

In addition to managing depression symptoms, preclinical studies suggest that antidepressants may inhibit glioblastoma progression and may be associated with improved outcomes. Many preclinical studies have underscored the complex interactions of neurotransmitters, specifically those implicated in antidepressant therapy, with glioblastoma and its effect on tumor proliferation.^3,4^ Several preclinical studies have identified Selective Serotonin Reuptake inhibitors (SSRIs), monoamine oxidase inhibitors (MOAIs), and tricyclic agents (TCAs) commonly used in the treatment of depression as having strong antiglioblastoma effects in both cell culture and mice models.^3,5–8^

Ultimately, the effect of antidepressants on glioblastoma survival is unknown and existing results from existing studies are conflicting.^9,10^ Thus, the effects of antidepressant therapy on large patient populations remains understudied.^11^

## Objective

We sought to perform a systematic review of the literature for any prospective, retrospective, or RCT studies that investigated the effect of antidepressant therapy on glioblastoma survival and to perform a meta-analysis of these results. In doing so, we hope to better understand the true benefit of antidepressant therapy in the treatment of glioblastoma.

## Methods

### Search Strategy and Information Sources

We performed a systematic review and meta-analysis according to a predetermined protocol registered on the International Platform of Registered Systematic Reviews and Meta-Analysis Protocols (INPLASY) (Registration Number INPLASY202470040, Supplementary eAppendix 1). This article was written in accordance to the Preferred Reporting Items for Systematic Reviews and Meta-Analyses guidelines.^11^ With the assistance of a trained librarian, we searched PubMed, Embase, Scopus, PsycINFO, and Web of Science from inception to March 2024 for articles describing glioblastoma and antidepressant therapy. Full search strategy including terms can be found in Supplementary eAppendix 2.

### Eligibility Criteria

Exclusion criteria included case reports, pilot reports, opinion pieces, theses, conference proceedings, letters, editorials, meta-analyses, reviews, surgical technique papers, abstracts, presentations, and non-English language publications without translation. Inclusion criteria included any observational or randomized control trials on adult patients with glioblastoma who was treated with antidepressants at any time after their glioblastoma diagnosis.

### Selection Process

Covidence systemic review software was utilized to aid in article screening. Duplicates were automatically removed by Covidence software.^12^ After the removal of duplicates, two reviewers (YS and MH) screen articles for inclusion criteria via title and abstract screening. After exclusion of articles, full-text screening was conducted by both authors to identify articles eligible for data extraction. Disagreements at any stage between reviewers were resolved by consensus.

### Data Collection Process

Two authors (YS and MH) independently extracted data from all articles that met eligibility for data collection using an excel data collection sheet. Data extracted included study characteristics, demographic data of the study population, effect size, overall survival, and other outcome measures. Secondary outcome information, such as progression free survival, was also extracted if available.

### Outcome

The primary outcome of interest was overall survival after glioblastoma diagnosis. Secondary outcomes were progression free survival.

### Synthesis Methods

We expected variability in patient selection among the studies. Therefore, we utilized a random‐effects model (REM) with restricted maximum-likelihood (REML) estimation.^13^ Prediction bands were calculated utilizing the Kenward Roger method.^14^ We utilized an inconsistency index (I^2^) to assess for heterogeneity. We calculated the mean difference in survival between treatment groups when applicable. Sensitivity analysis was conducted via the leave-one-out method and with Copas-like selection model (Supplementary eAppendix 3 and 4). Bias was assessed visually using funnel plots and via the Egger test (Supplementary eAppendix 5).^15^ All statistical analyses were performed using R (version 4.3.1).^16^ Packages utilized included the meta package.^17^ Alpha was set at 0.05, and all test of significance were 2-sided.

### Study Risk of Bias and Study Quality Assessment

Risk of bias was independently determined by the authors (YS and MH) for each study via the ROBINS-I tool (Supplementary eAppendix 7).^18^ Quality was assessed via the GRADE system and by assessing compliance to research reporting guidelines such as STROBE (Supplementary eAppendix 7 and 8).^19,20^ Competing interests in each study were if any author had ties to industry particularly those funded by an industry sponsor. There were none noted. Any disagreements were resolved via consensus or consultation with a third author (DEO).

## Results

### Study Selection

**Figure 1.**
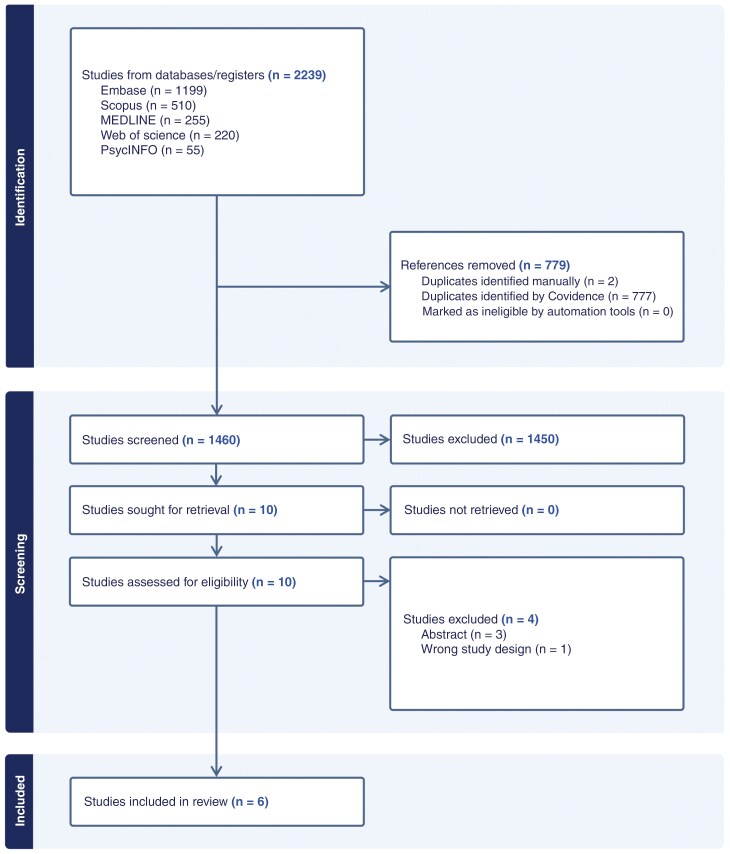
PRISMA flow diagram.

A total of 2,239 studies underwent abstract and title screening. Of this, 779 duplicates were removed. Of these, 1,450 articles that did not meet the inclusion criteria were excluded. Ten studies were sought for retrieval and underwent full-text review. Of these, 4 studies were excluded, 3 studies due to being abstracts, and 1 study due to having the wrong study design. This left 6 articles for inclusion. The results of our process are detailed as a PRISMA flow diagram in Figure 1.^21^

### Study Characteristics

Between the 6 studies, a total of 8,269 patients were included in our meta-analysis. Of these, 1,093 (13%) patients were treated for depression or depression symptoms at some point after their glioblastoma diagnosis. All 6 studies were retrospective cohort studies. One study used a national registry, and one study utilized pooled patients from three ongoing clinical trials. All studies included were conducted in Europe or the United States. Four of the 6 studies specified SSRI vs non-SSRI in their analysis, while 2 of the 6 studies included all antidepressant therapies in their analysis.

### Results of Individual Studies

**Figure 2.**
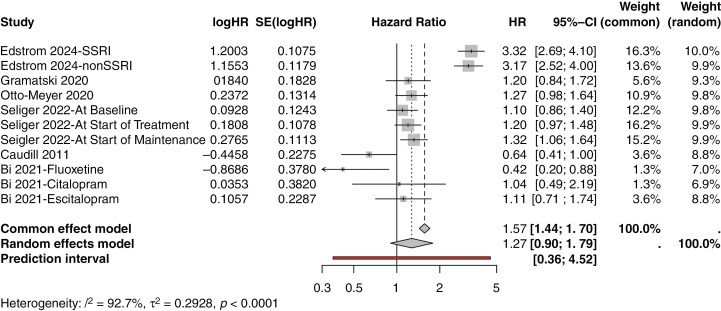
Meta-analysis results for overall survival.

**Figure 3.**
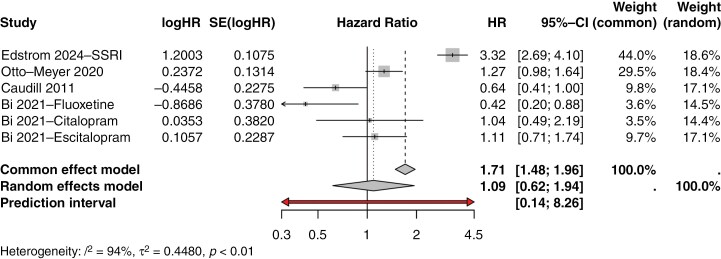
Subgroup meta-analysis results for SSRIs.

Studies were varied in their results, with 2 (33%) studies finding worsened survival associated with antidepressant therapy, 2 (33%) studies found survival benefit associated with antidepressant therapy, and 2 (33%) studies found nonstatistically significant results. Edstrom et al.^22^ found SSRI therapy to be associated with worse overall survival (HR 3.32, 95% CI 2.69–4.1, *P* < .001) and found non-SSRI antidepressant therapy to also be associated with worse overall survival (HR 3.54, 95% CI 2.52–4.0, *P* < .001). Gramatski et al.^23^ found antidepressant usage to trend towards with worsened overall survival (HR 1.2, 95% CI 0.84–1.72). Otto-Meyer et al.^9^ reported similar results (HR 1.27, 95% CI 0.98–1.64), though their analysis also did not reach statistical significance. Seliger et al.^10^ found baseline antidepressant use at time of beginning treatment for glioblastoma (HR 1.2, 95% CI 0.97–1.48, *P* = .038) and at during the start of maintenance cycle 4 of their clinical trial (HR 1.32, 95% CI 1.06–1.64, *P* = .003) to be associated with worsened survival. Caudill et al.^24^ found SSRI therapy to be associated improved overall survival (HR 0.67, 95% CI 0.2–0.88, *P* = .05). Bi et al.^25^ reported similar results, finding fluoxetine treatment to be associated with improved overall survival (HR 0.42, 95% CI 0.2–0.88, *P* = .022). Summaries of the included studies can be found in Table 1. After pooling and meta-analysis utilizing a random effects model with REML estimate, the generalized HR was 1.27 (95% CI 0.90–1.79, I^2^ = 93%, τ^2^ = 0.293) (Figure 2). After subanalysis for the effect of SSRI therapy alone, the generalized HR was 1.08 (0.58–2.03 I^2^ = 94%, τ^2^ = 0.55) (Figure 3).

**Table 1.** Summary of Studies Meeting Inclusion Criteria.

Author	Year	Study type	Study start year–End year	Sample size	Gender (M:F)	Drug exposure	Treatment size:Control size	Median age (range)	HR (95% CI)
Edstrom et al.	2024	Cohort	2009–2013	754	460:294	SSRI; MOI; TCA Mianserin; Mirtazapine; Bupropion; SNRI	549:205	63 (33–89)	Grade 4: 3.32 (2.69–4.10) Grade 2/3: 3.26 (2.19–4.85)
Gramatzki et al.	2020	Cohort	2005–2014	404	256:148	SSRI; SNRI; Mirtazapine	339:65	62 (18–90)	1.2 (0.84–1.72)
Otto-Meyer et al.	2020	Cohort	2000–2018	497	299:198	SSRI	346:151	64 (18–90)	1.27 (0.98–1.64)
Seliger et al.	2022	Cohort	2014–2017	1731	1026:705	SSRI; SNRI; TCA; Tranylcypromine; Moclobemide; Mianserin; Mirtazapine; Maprotiline; Lithium; Agomelatine; Bupropion; Trazodone; Tianeptine	146:1585	57 (18–90)	1.32 (1.06–1.64)
Caudill et al.	2011	Cohort	1999–2008	160	104:56	SSRI	125:35	58 (3–87)	0.67 (0.41–1.00)
Bi et al.	2021	Cohort	2003–2017	238	138:100	Fluoxetine	182:56	56 (23–80)	0.42 (0.20–0.88)
Bi et al.	2021	Cohort	2003–2017	238	138:100	Citalopram	182:56	56 (23–80)	1.03 (0.49–2.19)
Bi et al.	2021	Cohort	2003–2017	238	138:100	Escitalopram	182:56	56 (23–80)	1.11 (0.71–1.74)

SNRI = serotonin-norepinephrine reuptake inhibitors; SSRI = selective serotonin reuptake inhibitors; MOI = monoamine oxidase inhibitors; TCA = tricyclic antidepressants.

### Bias Analysis

We conducted an analysis of publication bias via Eggers’s method and funnel plot analysis, which was not significant (*P* = .122) (Supplementary eAppendix 5).^15^ Risk of bias was investigated using the ROBINS-I tool.^18^ Two (33%) of the studies were found to have moderate overall bias. However, 4 (66.7%) of the 6 studies were found to have serious bias (Supplementary eAppendix 6). Studies by Otto-Meyer et al.,^9^ Caudill et al.,^24^ and Bi et al.^25^ were found to have “serious” bias in the domain of bias due to confounding. This was assessed due to failure to adjust for molecular and genetic testing data in glioblastoma, namely MGMT and IDH mutation status, that have been found to significantly predict outcomes for glioblastoma.^26,27^ Serious bias was assessed in the classification of interventions domain in the study conducted by Seliger et al.^10^ due to the patients’ involvement in an ongoing trial. Thus, it is difficult to assess the true effect of antidepressant therapy in this population. Bias was also assessed via analysis of adherence to reporting guidelines. Certainty of evidence assessment can be found in Supplementary eAppendix 7. As observational cohort studies, all studies adhered to Strengthening the Reporting of Observational Studies in Epidemiology (STROBE) guidelines.^19^ Two of the studies met 21/22 items of the guideline, 2 studies met 20/22 items, and 2 studies met 22/22 items of the guideline (Supplementary eAppendix 8).

### Sensitivity Analysis

We conducted sensitivity analysis via the leave-one-out method. The results of this analysis are found in Supplementary eAppendix 3. We also performed a sensitivity analysis taking into account assumed publication bias via the copas selection model.^28^ The results of this analysis are found in Supplementary eAppendix 4.

## Discussion

Depression is a common comorbidity of glioblastoma.^29^ The presence of depression in glioblastoma can significantly impact quality of life and outcomes. Potential mechanisms by which this may occur is through decreasing adherence to medication regimens, impairment of function and activities of daily living, lowered overall quality of life, and may lead to increased rate of deterioration in patients.^2^ This pooled analysis of 6 studies suggests that the administration of antidepressants in patients with glioblastoma is associated with worse overall survival.

These results are somewhat surprising given the volume of preclinical evidence in support of the utilization of antidepressant therapy. The use of antidepressants on depression in glioblastoma patients may improve patient function and lead to improved outcomes. However, there are other pathways that may contribute to improved function. Studies have suggested a complex interaction between glioblastoma and neurons driving tumor proliferation.^30,31^ Though still poorly understood, antidepressants may disrupt such signaling and lead to slower tumor progression.

Furthermore, several studies suggest antidepressants alter the NFKB pathway and mTOR pathways, inducing apoptosis in glioblastoma cells through a variety of mechanisms.^32,33^ Several studies have shown that antidepressant treatment can decrease the invasiveness of glioblastoma and induce autophagy in others.^34^ Thus, some studies suggest that the suppression of these transcription factors that are highly associated with glioblastoma progression would be beneficial as adjuvant therapy in addition to standard of care. These results are also surprising given that depression is a known comorbidity in patents with glioblastoma.^2,35^ Suggested mechanism by which this process occurs involve brain-derived neurotrophic factor, with some studies suggesting that the dysregulation of BDNF in glioblastoma may contribute to depressive symptoms.^36^ Other studies have suggested that pathologic changes in the serotonin pathway may play a role in glioblastoma and depression as well.^37^ It has been well established that depression is associated with worse survival in almost all forms of cancer.^38^ The same results have been seen in glioblastoma, with an analysis by Shi et al.^39^ finding a difference of −0.56 months for glioma patients with depression. Furthermore, inflammation induced by depression may increase the rate of cognitive decline and lead to worse overall survival.^40,41^ Thus, it stands to reason that the treatment of this depression should improve outcomes.

Our results found that, in a pooled analysis, administration of antidepressants for patients with glioblastoma trended towards worse overall survival, though this was not statistically significant (Figure 2). With this, we may reasonably suggest that while antidepressant therapy is safe for administration in patients with glioblastoma, it does not significantly improve outcomes. Thus, despite potential improvements in quality of life and the alleviation of depression symptoms, antidepressant therapy may not increase overall survival.

However, several factors should be included when considering these results. Studies have found that patients with glioblastoma and depression have glioblastoma cells that are more necrotic, have increased proliferation, and peritumor edema. These patient’s cells also have greater invasiveness in preclinical studies.^42^ This may suggest that patients with glioblastoma and depression may have more severe phenotypes of tumor, leading to confounding of any survival benefit gained from antidepressant therapy. Furthermore, other studies have found that antidepressant therapy with substances such as imipramine and tranylcypromine may interfere with the cytotoxicity of temozolomide, currently included as standard of care as part of the Stupp protocol, an effect not seen in fluoxetine.^43,44^ This may be explained why a survival benefit was observed with fluoxetine analysis by Bi et al.,^25^ but not any other SSRIs. Other studies have also shown that SSRIs lower the seizure threshold.^45^ As seizures commonly manifest in patients with glioblastoma, potentially more common and severe seizures in glioblastoma patients on SSRIs may lead to decreased quality of life and ultimately worse survival overall, despite any decrease in glioblastoma progression.^46^

Another important factor to consider in these results is the issues with available literature itself. In our sensitivity analysis, the true effect of antidepressants therapy on glioblastoma survival remains unclear. The high heterogeneity makes it difficult to draw a definitive conclusion from existing literature. The differences between studies may be attributed to several factors. Variations in the definition of antidepressants between studies also limits the meta-analysis. The included studies rarely defined what type of antidepressant was utilized, and only one study (16.7%) stratified to compare the efficacies of different antidepressants. Though a category defined by clinical use, antidepressants can vary widely in chemical structure and mechanism of action, thus leading to a diverse array of potential effects on glioblastoma tumor proliferation in patients. Though we attempted to investigate this with a subgroup analysis of studies that only included SSRIs, some studies did not stratify between SSRIs and non-SSRIs, limiting our subgroup analysis. Studies had limited data with regards to timing of antidepressant therapy, dosage, and other medications that may interfere with medication metabolism. The study conducted by Siegler et al.^10^ included in our meta-analysis may also confound our data, as they included patients not on current standard of care, recruiting instead from an existing registry of clinical trial patients from the CENTRIC, CORE, ACT-IV, and AVAGlio clinical trials. The addition of the novel antiglioblastoma agents in combination with standard therapy may confound any observed effects of antidepressants. The study by Edstrom et al.^22^ utilized a registry-based population spread across multiple centers. The difference in standards of care or socio-environmental factors for glioblastoma patients across different centers and heterogenous treatment population may confound the results as well.

### Limitations

This meta-analysis is limited by the heterogeneity of the studies. Furthermore, all the studies included were observational studies, which are considered low quality of evidence by the GRADE system. Furthermore, several studies did not include biomolecular markers of glioblastoma, such as MGMT methylation and IDH mutation status, with their proportional hazards models, thus limiting the applicability of their results. Pooled analyses of the data also limit the interpretation of the results, as individual patient data would be beneficial in allowing a more accurate analysis of the effect size.

## Conclusions

Our results show that while antidepressant therapy for patients with glioblastoma does not improve overall survival, it is not associated with worsened outcomes and may be safely initiated in patients with glioblastoma. However, further studies with higher quality evidence and incorporation of new biomolecular data warrant investigation.

## Supplementary Material

vdaf075_suppl_Supplementary_Appendix

## Data Availability

Data and syntax for this analysis are publicly available through GitHub.

## References

[1] Grochans S , Cybulska AM, Simińska D, et al Epidemiology of glioblastoma multiforme-literaturereview. Cancers (Basel) 2022;14(10):2412. 10.3390/cancers14102412 PMC9139611 35626018

[2] van der Meer PB , Dirven L, Hertler C, et al Depression and anxiety in glioma patients. Neurooncol Pract. 2023;10(4):335–343. 37457222 10.1093/nop/npad019 PMC10346395

[3] Liu KH , Yang S-T, Lin Y-K, et al Fluoxetine, an antidepressant, suppresses glioblastoma by evoking AMPAR-mediated calcium-dependent apoptosis. Oncotarget 2015;6(7):5088–5101. 25671301 10.18632/oncotarget.3243 PMC4467135

[4] Chen VC-H , Hsieh Y-H, Chen L-J, Hsu T-C, Tzang B-S. Escitalopram oxalate induces apoptosis in U-87MG cells and autophagy in GBM8401 cells. J Cell Mol Med. 2018;22(2):1167–1178. 29105282 10.1111/jcmm.13372 PMC5783874

[5] Higgins S , et al P08. 57 Involvement of both the extrinsic and intrinsic apoptotic pathways with clomipramine treatment of human glioblastoma cells under normoxic and hypoxic conditions. Neuro-Oncology. 2016;18(4):iv54.

[6] Kast RE , Karpel-Massler G, Halatsch M-E. CUSP9* treatment protocol for recurrent glioblastoma: aprepitant, artesunate, auranofin, captopril, celecoxib, disulfiram, itraconazole, ritonavir, sertraline augmenting continuous low dose temozolomide. Oncotarget. 2014;5(18):8052. 25211298 10.18632/oncotarget.2408 PMC4226667

[7] Parker KA , Pilkington GJ. Apoptosis of human malignant glioma-derived cell cultures treated with clomipramine hydrochloride, as detected by Annexin-V assay. Radiol Oncol. 2006;40(2):87.

[8] Troib A , Azab A. Effects of psychotropic drugs on nuclear factor kappa B. Eur Rev Med Pharmacol Sci. 2015;19(7):1198. 25912579

[9] Otto-Meyer S , DeFaccio R, Dussold C, et al A retrospective survival analysis of Glioblastoma patients treated with selective serotonin reuptake inhibitors. Brain Behav Immun Health 2015;2:100025. 10.1016/j.bbih.2019.100025 PMC7079579 32190845

[10] Seliger C , Oppong FB, Lefranc F, et al; EORTC Brain Tumor Group. Association of antidepressant drug use with outcome of patients with glioblastoma. Int J Cancer. 2023;152(7):1348–1359. 36346112 10.1002/ijc.34344

[11] Page MJ , McKenzie JE, Bossuyt PM, et al The PRISMA 2020 statement: an updated guideline for reporting systematic reviews. bmj 2021;372:n71. 33782057 10.1136/bmj.n71 PMC8005924

[12] Babineau, J. Product review: Covidence (systematic review software). J Can Health Libraries Assoc/Journal de l’Association des bibliothèques de la santé du Canada 2014;35(2):68–71.

[13] Dongen SV. Molenberghs & Matthysen. The statistical analysis of fluctuating asymmetry: REML estimation of a mixed regression model. J Evol Biol. 1999;12(1):94–102.

[14] Kenward MG , Roger JH. The use of baseline covariates in crossover studies. Biostatistics 2009;11(1):1–17. 19915170 10.1093/biostatistics/kxp046

[15] Egger M , Smith GD, Schneider M, Minder C. Bias in meta-analysis detected by a simple, graphical test. Bmj 1997;315(7109):629–634. 9310563 10.1136/bmj.315.7109.629 PMC2127453

[16] Team, R. C. R: A Language and Environment for Statistical Computing. Vienna, Austria: Team, R. C.; 2022.

[17] Schwarzer G. An R package for meta-analysis. R News 2007;7:40–45.

[18] Sterne JA , Hernán MA, Reeves BC, et al ROBINS-I: a tool for assessing risk of bias in non-randomised studies of interventions. BMJ 2016;355:i4919. 27733354 10.1136/bmj.i4919 PMC5062054

[19] Vandenbroucke JP , von Elm E, Altman DG, et al; STROBE Initiative. Strengthening the reporting of observational studies in epidemiology (STROBE): explanation and elaboration. Int J Surg. 2014;12(6):1500–1524. 25046751 10.1016/j.ijsu.2014.07.014

[20] Guyatt GH , Oxman AD, Vist GE, et al; GRADE Working Group. GRADE: an emerging consensus on rating quality of evidence and strength of recommendations. Bmj 2020;336:924–926. 10.1136/bmj.39489.470347.AD PMC2335261 18436948

[21] Salameh JP , Bossuyt PM, McGrath TA, et al Preferred reporting items for systematic review and meta-analysis of diagnostic test accuracy studies (PRISMA-DTA): explanation, elaboration, and checklist. BMJ 2008;370:m2632. 10.1136/bmj.m2632 32816740

[22] Edström S , Hellquist BN, Sandström M, et al Antidepressants and survival in glioma—a registry-based retrospective cohort study. Neurooncol. Pract.. 2023;11(2):125–131. 38496917 10.1093/nop/npad057 PMC10940821

[23] Gramatzki D , Rogers JL, Neidert MC, et al Antidepressant drug use in glioblastoma patients: an epidemiological view. Neurooncol. Pract.. 2020;7(5):514–521. 33014392 10.1093/nop/npaa022 PMC7516105

[24] Caudill JS , Brown PD, Cerhan JH, Rummans TA. Selective serotonin reuptake inhibitors, glioblastoma multiforme, and impact on toxicities and overall survival: the mayo clinic experience. Am J Clin Oncol. 2011;34(4):385–387. 20859197 10.1097/COC.0b013e3181e8461a

[25] Bi J , Khan A, Tang J, et al Targeting glioblastoma signaling and metabolism with a re-purposed brain-penetrant drug. Cell Rep. 2021;37(5):109957. 34731610 10.1016/j.celrep.2021.109957 PMC8856626

[26] Weller M , et al Molecular predictors of progression-free and overall survival in patients with newly diagnosed glioblastoma: a prospective translational study of the German Glioma Network. JCO 2009;27(34):5743–5750. 10.1200/JCO.2009.23.0805 19805672

[27] Balss J , Meyer J, Mueller W, et al Analysis of the IDH1 codon 132 mutation in brain tumors. Acta Neuropathol. 2008;116(6):597–602. 18985363 10.1007/s00401-008-0455-2

[28] Copas JB , Li HG. Inference for non-random samples. J R Stat Soc. 2002;59(1):55–95. doi: https://doi.org/10.1111/1467-9868.00055.

[29] Mugge L , Mansour TR, Crippen M, Alam Y, Schroeder J. Depression and glioblastoma, complicated concomitant diseases: a systemic review of published literature. Neurosurg Rev. 2020;43(2):497–511. 30094499 10.1007/s10143-018-1017-2

[30] Krishna S , Choudhury A, Keough MB, et al Glioblastoma remodelling of human neural circuits decreases survival. Nature. 2023;617:599–607. 37138086 10.1038/s41586-023-06036-1 PMC10191851

[31] Venkatesh HS , Morishita W, Geraghty AC, et al Electrical and synaptic integration of glioma into neural circuits. Nature. 2019;573:539–545. 31534222 10.1038/s41586-019-1563-y PMC7038898

[32] Hsu F-T , Chiang I-T, Wang W-S. Induction of apoptosis through extrinsic/intrinsic pathways and suppression of ERK/NF-κB signalling participate in anti-glioblastoma of imipramine. J Cell Mol Med. 2020;24(7):3982–4000. 32149465 10.1111/jcmm.15022 PMC7171418

[33] Hayashi K , Michiue H, Yamada H, et al Fluvoxamine, an anti-depressant, inhibits human glioblastoma invasion by disrupting actin polymerization. Sci Rep. 2016;6:23372. 26988603 10.1038/srep23372 PMC4796892

[34] Dikmen M , Cantürk Z, Öztürk Y. Escitalopram oxalate, a selective serotonin reuptake inhibitor, exhibits cytotoxic and apoptotic effects in glioma C6 cells. Acta Neuropsychiatrica 2011;23(4):173–178. 25379795 10.1111/j.1601-5215.2011.00550.x

[35] Young JS , Al-Adli N, Sibih YE, et al Recognizing the psychological impact of a glioma diagnosis on mental and behavioral health: a systematic review of what neurosurgeons need to know. J Neurosurg. 2022;139(1):11–19. 36334288 10.3171/2022.9.JNS221139 PMC10413205

[36] Colucci-D’Amato L , Speranza L, Volpicelli F. Neurotrophic factor BDNF, physiological functions and therapeutic potential in depression, neurodegeneration and brain cancer. Int J Mol Sci . 2020;21(20):7777. 33096634 10.3390/ijms21207777 PMC7589016

[37] Merzak A , Koochekpour S, Fillion M-P, Fillion G, Pilkington GJ. Expression of serotonin receptors in human fetal astrocytes and glioma cell lines: a possible role in glioma cell proliferation and migration. Brain Res Mol Brain Res. 1996;41(1–2):1–7. 8883928 10.1016/0169-328x(96)00058-7

[38] Walker J , Mulick A, Magill N, et al Major depression and survival in people with cancer. Psychosom Med. 2021;83(5):410–416. 33938501 10.1097/PSY.0000000000000942 PMC7614901

[39] Shi C , Lamba N, Zheng LJ, et al Depression and survival of glioma patients: a systematic review and meta-analysis. Clin Neurol Neurosurg. 2018;172:8–19. 29957299 10.1016/j.clineuro.2018.06.016

[40] Ting EY , Yang AC, Tsai SJ. Role of interleukin-6 in depressive disorder. Int J Mol Sci . 2020;21(6):2194. 32235786 10.3390/ijms21062194 PMC7139933

[41] Postal M , Lapa AT, Sinicato NA, et al Depressive symptoms are associated with tumor necrosis factor alpha in systemic lupus erythematosus. J Neuroinflammation. 2016;13:5. 26732584 10.1186/s12974-015-0471-9 PMC4702302

[42] Fu X , Wu C, Han N, et al Depressive and anxiety disorders worsen the prognosis of glioblastoma. Aging (Albany NY) 2020;12(20):20095–20110. 33113511 10.18632/aging.103593 PMC7655183

[43] Bielecka AM , Obuchowicz E. Antidepressant drugs can modify cytotoxic action of temozolomide. Eur J Cancer Care (Engl). 2017;26(5). 10.1111/ecc.12551 27480195

[44] Stupp R , Mason WP, van den Bent MJ, et al; European Organisation for Research and Treatment of Cancer Brain Tumor and Radiotherapy Groups. Radiotherapy plus concomitant and adjuvant temozolomide for glioblastoma. N Engl J Med. 2005;352(10):987–996. 15758009 10.1056/NEJMoa043330

[45] Jiménez-Jiménez FJ , Puertas I, de Toledo-Heras M. Drug-induced myoclonus. CNS Drugs 2004;18(2):93–104. 14728056 10.2165/00023210-200418020-00003

[46] Vecht CJ , Kerkhof M, Duran-Pena A. Seizure prognosis in brain tumors: new insights and evidence-based management. Oncologist. 2014;19(7):751–759. 24899645 10.1634/theoncologist.2014-0060 PMC4077452

