# Supplementary material for: The effect of antidepressants on glioblastoma survival: A systematic review and meta-analysis
Source: Neurooncol Adv. 2025 Jun 14;7(1):vdaf075. doi: 10.1093/noajnl/vdaf075 (PMC12224260; doi:10.1093/noajnl/vdaf075)
Supplement: vdaf075_suppl_Supplementary_Appendix [file vdaf075_suppl_supplementary_appendix.docx]

**Supplemental Materials and Data**

**eAppendix 1**. Study design and protocol as registered with International Platform of Registered Systematic Review and Meta-analysis Protocols (INPLASY) (registration number INPLASY202470040).

V2.0

Edits: Adjustments in author list

**Effect of Antidepressants on Glioblastoma Survival: A Systematic Review and Meta-analysis**

**Title Page**

**Authors:**

Yifei Sun, BS

Mohammad Hamo, BM

Travis K Atchley, MD

Burt Nabors, MD

James Markert, MD MPH

Dagoberto Estevez-Ordonez, MD-PhD

Study Contributions:

**Contributions of each author:**

Author 1 – Yifei Sun – Study design, analysis, guarantor of review, statistical analysis, data extraction, manuscript writing and review

Email: [ysun3@uab.edu](mailto:ysun3@uab.edu)

Author 2 – Mohammad Hamo - Study design, analysis, guarantor of review, statistical analysis, data extraction, manuscript writing and review

Email: [mahamo@uab.edu](mailto:mahamo@uab.edu)

Author 3 – Dagoberto Estevez-Ordonez- Study design, analysis, guarantor of review, statistical analysis, manuscript writing and review

Email: [destevezordonez@uabmc.edu](mailto:destevezordonez@uabmc.edu)

Author 4 - Travis Atchley – Study design, analysis, manuscript writing and review

Email: [tactchley@uabmc.edu](mailto:tactchley@uabmc.edu)

Author 4 – Burt Nabors – Oversight of design, manuscript writing, review, and supervision

Email: [bnabors@uabmc.edu](mailto:bnabors@uabmc.edu)

Author 5 – James M Markert - Oversight of design, manuscript writing, review, and supervision

Email: [jmarkert@uabmc.edu](mailto:jmarkert@uabmc.edu)

Corresponding Author:

Dagoberto Estevez-Ordonez MD PhD

destevezordonez@uabmc.edu

Chief Resident

Department of Neurosurgery, University of Alabama at Birmingham, Alabama

Funding/Support:

This project is supported in part by the National Institute of Neurological Disorders and Stroke of the National Institutes of Health under award number R25NS079188 (DEO). The content is solely the responsibility of the authors and does not necessarily represent the official views of the National Institutes of Health. DEO is also a Cornwall Clinical Scholar supported by the University of Alabama at Birmingham.

**Introduction**

Rationale:

Despite advancements in the treatment of GBM, prognosis remains poor. Symptoms of depression are highly prevalent among patients with GBM and have been associated with poor outcomes. However, much of literature is inconclusive on whether the treatment of depression symptoms in GBM patients improves outcomes. Furthermore, there exist concerns that antidepressant may negatively GBM outcomes through downregulation of tumor inhibitor pathways.

In addition to managing depression symptoms, preclinical studies suggest that antidepressants may inhibit GBM progression and may be associated with improved outcomes. Several preclinical studies have identified Selective Serotonin Reuptake inhibitors (SSRIs), monoamine oxidase inhibitors (MOAIs), and tricyclic agents commonly used in the treatment of depression as having strong anti-GBM effects in both cell culture and mice models.^3,5-8^

Ultimately, depression rates are disproportionately high in patients with GBM. The treatment of depression in these patients may have improved survival. Preclinical studies also suggest that antidepressant therapy may improve survival via inhibition of growth receptors and other signaling pathways. However, the effect of antidepressants on survival is unknown and results from existing studies are conflicting, with several recent retrospective studies reporting improved survival and others reporting either no effect or a detrimental effect on outcome. ^9,10^

We sought to perform a systematic review of the literature for any prospective, retrospective, or RCT studies that investigated the effect of antidepressant therapy on GBM survival and to perform a meta-analysis of these results. In doing so, we hope to better understand the true benefit of antidepressant therapy in the treatment of GBM.

Aims:

*1*. Determine if Antidepressant therapy initiated before or during standard treatment (maximal resection followed by radiation and adjuvant chemotherapy) for adults with Glioblastoma (GBM) improves overall survival (OS) when compared to patients not on antidepressant therapy.

Research Question

*Research Question:*

Patients:

- Adult patients (>18) with histologically/biospy confirmed GBM

Intervention:

- Antidepressant therapy in addition to standard of care

Comparator:

- Standard of care without antidepressant therapy

Outcomes

- Primary Outcome: Overall survival

**Condition Being Studied:** Glioblastoma (GBM) is the most common primary malignant tumor of the brain, comprising nearly 50% of all central nervous system tumors. Despite improvements in care, it continues to carry a poor prognosis, with 5-year survival rates under 7% and an average survival time of 8 months post diagnosis. Depression is a common comorbidity of patients with GBM, with some studies suggesting rates ranging from 33% to 44%.^11^ Consequently, studies have shown that depression is associated with worsened outcomes in GBM. However, the effects of antidepressant therapy on GBM patient populations remains understudied and poorly understood.

**Methods/Approach**

Study Design:

Systematic review and meta-analysis of literature adhering to the PRISMA checklist guideline ^12,13^

**Eligibility criteria:**

*Study exclusion criteria:*

Case reports, pilot reports, opinion pieces, theses, conference proceedings, letters, editorials, meta-analysis, reviews, surgical technique papers, abstracts, presentations, and non-english language publications without translation

**Information Sources:**

PubMed, Embase, Scopus, PsycINFO, Web of Science

Search Strategy:

Concept 1 (Antidepressant Agents) AND Concept 2 (Glioblastoma) NOT ('animal'/exp NOT 'human'/exp)

(('antidepressant agent'/exp OR (anti-depress* OR antidepress* OR neurothymoleptic OR psychoenergizer OR thymoleptic OR thymolytic-agent):ti,ab,kw) OR ('serotonin uptake inhibitor'/de OR 'serotonin noradrenalin reuptake inhibitor'/de OR 'monoamine oxidase inhibitor'/exp OR (SSRI* OR SNRI* OR SSNRI* OR ((serotonin OR 'dual uptake' OR 'dual reuptake' OR noradrenalin OR monoamine OR dopamine OR norepinephrine OR MOA) NEAR/2 (inhibit*))):ti,ab,kw) OR ('citalopram'/de OR (acelopam OR adeprenal OR apo-cital OR aurex OR ceform OR celexa OR cilopress OR cinavol OR ciprager OR cipram OR cipramil OR cipraned OR ciprotan OR ciral OR citabax OR citacip OR citagen OR cital* OR citalon* OR citalopram OR citalostad OR citalox OR citalvir OR citapram OR citaxin OR citesint OR citopam OR citrol OR citronil OR cytalopram OR dalsan OR elopram OR exenadil OR frimaind OR futuril OR galopran OR humorap OR kaidor OR kitapram OR linisan OR lopracil OR lopraxer OR loxopram OR lupram OR malicon OR nitalapram OR oropram OR percitale OR pralotam OR pramital OR prefucet OR pricital OR prisdal OR psiconor OR renevil OR ricap OR ropramin OR selon OR sepram OR seralgan OR seregra OR serital OR seropram OR seror OR sintopram OR sotovon OR talam OR talosin OR varom OR vesema OR xadorek OR zanipram OR zeclicid OR zentius OR zitolex OR zyloram):ti,ab,kw) OR ('escitalopram'/de OR (aciprex OR amasci OR anxila OR apoescitaxin OR betesda OR cipralex OR citafort OR citram OR clomentin OR depralin OR depresinal OR diprex OR ecytara OR elicea OR entact OR escidivule OR escipram OR esciprex OR escirdec OR escital* OR escitalopram OR escitalpro OR escitil OR esertia OR eslorex OR esoprex OR espoza OR estan OR etalopro OR giachela OR heipram OR itakem OR lenuxin OR lexapro OR mersinol OR miraklide OR mozarin OR nexpram OR oroes OR pralex OR pramatis OR prameffex OR pramulex OR premalex OR prilect OR raldon OR ratice OR scippa OR serodeps OR seroplex OR serpentil OR servenon OR sipralexa OR symescital OR tepram OR zecidec OR zocital):ti,ab,kw) OR ('fluoxetine'/de OR (Actan OR adofen OR afeksin OR andep* OR animex-on OR ansilan OR auroken OR auscap OR bioxetin OR captaton OR daforin OR dagrilan OR depren OR deprex-leciva OR deprexetin OR deprexin OR deprizac OR deproxin OR diesan OR digassim OR elizac OR eufor OR exostrept OR felicium OR fldiss OR flotinal OR floxet OR fluctin* OR fludac OR flufran OR fluketin OR flunil OR flunirin OR fluocim OR fluohexal OR fluoksetin OR fluoksetyna OR fluox OR fluoxac OR fluoxeren OR fluoxetin* OR fluoxifar OR fluoxil OR fluoxin OR fluoxone OR fluoxtab OR fluronin OR flusac OR flustad OR flutin OR flutine OR fluxemed OR fluxen OR fluxet* OR fluxil OR fluxomed OR fluzac OR fluzak OR fokeston OR fontex OR foxetin OR foxtin OR fropine OR fuloren OR gerozac OR ladose OR lanclic OR lorien OR lovan OR luramon OR magrilan OR margrilan OR meropan OR modipran OR mutan OR nopres OR nuzak OR olena OR oxactin OR oxedep OR plazeron OR plinzene OR pragmaten OR prizma OR proctin OR prodep OR prosac OR prozac OR prozamel OR prozamin OR prozep OR prozit OR psipax OR psiquial OR qualisac OR rapiflux OR reconcile OR reneuron OR rowexetina OR salipax OR sanzur OR sarafem OR sartuzin OR selfemra OR serelsa OR seromex OR seronil OR sinzac OR sofelin OR tuneluz OR veritina OR xeredien OR zactin OR zepax OR zinovat):ti,ab,kw) OR ('paroxetine'/de OR

(arketis OR aropax OR aroxat OR brisdelle OR daparox OR deroxat OR dexorat OR divarius OR dropax* OR euplix OR eutimil OR frosinor OR mesafem OR motivan OR optipar OR paluxetil OR paluxon OR paroc OR parogen OR paroxedura OR paroxet* OR paxan OR paxil OR paxtine OR paxxet OR pexeva OR serestill OR sereupin OR seroxat OR setine OR syntopar OR tagonis OR paroxetine):ti,ab,kw) OR ('sertraline'/de OR (adjuvin OR ainim OR altisben OR altruline OR aremis OR asentra OR aserin OR asertin OR atruline OR besitran OR certorun OR contulen OR depreger OR dominum OR doxime OR enidap OR enore OR epilyd OR ferbrain OR fridep OR gladem OR lesefer OR lustral OR luxeta OR miravil OR neurosedine OR nudep OR sastium OR seltra OR semonic OR sercerin OR serimel OR serlain OR serlan OR serlift OR serlin OR serolux OR seromeg OR sertabal OR sertadepi OR sertagen OR sertral OR sertralet OR sertraline OR sertralon OR sertranat OR sertranex OR sertranorm OR sertranquil OR sertrone OR serunato OR setaloft OR sonalia OR sosser OR stimuloton OR tatig OR tresleen OR zolof OR zoloft OR zolotrin OR zortal OR zosert OR zotral):ti,ab,kw) OR ('desvenlafaxine'/exp OR (desvenlafaxine OR desmethylvenlafaxine OR ellefore OR khedezla OR norvenlafaxine OR Pristiq OR pristiqs):ti,ab,kw) OR ('duloxetine'/exp OR (ariclaim OR cymbalta OR drizalma OR dulane OR duloxetine OR duzela OR irenka OR nodetrip OR xeristar OR yentreve):ti,ab,kw) OR ('milnacipran'/exp OR (dalcipran OR fetzima OR impulsor OR ixel OR joncia OR levomilnacipran OR midalcipran OR milnacipran OR milnaneurax OR savella OR toledomin):ti,ab,kw) OR ('venlafaxine'/exp OR (alventa OR amphero OR apclaven OR 'arafaxina retard' OR axyven OR bonilux OR depefex OR deprevix OR dobupal OR duofaxin OR efaxine OR efectin OR efexor OR effexor OR effexstad OR elafax OR elify OR faxigen OR faxiprol OR faxiven OR faxolet OR fobiless OR genexin OR hapixed OR ireven OR lafactin OR lanvexin OR majoven OR olwexya OR oriven OR pracet OR prefaxine OR serosmine OR sigven OR sivion OR sunveniz OR symfaxin OR tonpular OR trevilor OR trewilor OR vandral OR vaxor OR vedixal OR velafax OR velaxin OR velept OR venaxx OR vencarm OR venex OR venla OR venlablue OR venlabrain OR venladex OR venlafab OR venlafaxin OR venlafaxina OR venlafaxine OR venlafex OR venlagamma OR venlalic OR venlaneo OR venlasov OR venlatev OR venlax OR venlaxin OR venlaxor OR venlazid OR venlectine OR venlofex OR venlor OR venprimeven OR vensir OR vensuerteven OR venxin OR venzip OR vexarin OR viepax OR xenalven OR zacalen OR zaredrop OR zarelis OR zarelix OR venlafaxine):ti,ab,kw) OR ('atypical antidepressant agent'/exp) OR ('amfebutamone'/exp OR (amfebutamone OR aplenzin OR budeprion OR buprion-hydrochloride OR bupropin OR bupropion OR buproprion OR buxon OR elontril OR forfivo OR odranal OR quomem OR quomen OR wellbatrin OR wellbutrin OR zyban):ti,ab,kw) OR ('mirtazapine'/exp OR (azamianserin OR afloyan OR avanza OR calixta OR depreram OR esprital OR mepirzapin OR mirap OR mirataz OR mirtabene OR mirtadepi OR mirtalich OR mirtamor OR mirtapil OR mirtastad OR mirtazapin* OR mirtel OR mirtin OR mirtor OR mirzaten OR mitabor OR mizapin OR norset OR pharmataz OR promyrtil OR psidep OR remergil OR remergon OR remeron OR remirta OR rexer OR saxib OR yarocen OR zismirt OR zispin):ti,ab,kw) OR ('nefazodone'/exp OR (dutonin OR nefadar OR nefazodone OR reseril OR serzone OR serzonil):ti,ab,kw) OR ('trazodone'/exp OR (azonz OR beneficat OR bimaran OR deprax OR depresil OR depyrel OR desirel OR desyrel OR manegan OR molipaxin OR oleptro OR pesyrel OR pragazone OR pragmarel OR pragmazone OR reslin OR taxagon OR thombran OR thromban OR thrombran OR tombran OR tradozone OR trasodon OR trasodone OR trazodil OR trazodon OR trazodone OR trazolan OR trialodine

OR trittico):ti,ab,kw) OR ('vilazodone'/exp OR (viibryd OR vilazodone):ti,ab,kw) OR ('vortioxetine'/exp OR (brintellix OR trintellix OR vortioxetine):ti,ab,kw) OR ('isocarboxazid'/exp OR (bmih OR enerzer OR isocarboazide OR isocarboxacid OR isocarboxazid* OR marplan):ti,ab,kw) OR ('phenelzine'/exp OR (fenelzine OR fenizin OR mao-rem OR nardelzine OR nardil OR phenalzine OR phenelzin* OR phenethylhydrazine OR phenylethylhydrazine OR stinerval):ti,ab,kw) OR ('selegiline'/exp OR (anipryl OR antiparkin OR apo-selegiline OR atapryl OR deprenaline OR deprenil OR deprenyl OR egibren OR eldeprine OR eldepryl OR elegelin OR emsam OR julab OR julegil OR jumex OR jumexal OR kinline OR l-deprenyl OR levo-deprenyl OR mao-b OR maotil OR movergan OR niar OR otrasel OR parkryl OR phenylisopropylmethylpropynylamine OR plurimen OR procythol OR sedicel OR sefmex OR seledat OR selegil OR selegiline OR selegos OR selgene OR selgin OR xilopar OR zelapar OR selegiline):ti,ab,kw) OR ('tranylcypromine'/exp OR (jatrosom OR parmodalin OR parnate OR parnitene OR parnitine OR trancilpromine OR trancylpromine OR trancylprominesulfate OR trancylprominesulphate OR tranilacipromina OR transamine OR tranylcypomia OR tranylcypromide OR tranylcypromin* OR tylciprine):ti,ab,kw) OR ('tricyclic antidepressant agent'/exp OR (tricyclic NEAR/3 antidepress*):ti,ab,kw) OR ('amitriptyline'/exp OR (adepress OR adepril OR ambivalon OR amilit OR amineurin OR amiplin OR amiprin OR amirol OR amitid OR amitril OR amitrip OR amitriptylene OR amitriptylin* OR amitriptylinumhydrochloride OR amitryptiline OR amitryptilline OR amitryptine OR amitriptyline OR amyline OR amytril OR amytriptiline OR amytriptyline OR amytryptiline OR amyzol OR anapsique OR antalin OR antitriptyline OR astilin OR damilen* OR damitriptyline OR damylene OR deprelio OR domical OR elatrol* OR elavil OR enafon OR endep OR enovil OR etafon OR etafron OR euplit OR lantron OR laroxal OR laroxyl OR lentizol OR miketorin OR novoprotect OR ormal OR pinsaun OR proheptadien OR qualitriptine OR redomex OR sarboten-retard OR sarotard OR saroten* OR sarotex OR stelminal OR sylvemid OR syneudon OR syneydon OR teperin OR terepin OR trepiline OR tridep OR tripta OR triptanol OR triptizol OR triptyl OR triptyline OR trynol OR tryptanol OR tryptizol OR trytomer OR uxen OR vanatrip):ti,ab,kw) OR ('amoxapine'/exp OR (adisen OR amoxan OR amoxapin* OR asendin OR asendis OR defanyl OR demolox OR moxadil):ti,ab,kw) OR ('desipramine'/exp OR (demethylimipramine OR deprexan OR desimipramine OR desipramin OR despiramine OR desipramine OR desmethyl-imipramin* OR desmethylimipramin* OR nebril OR norimipramine OR norpramin* OR nortimil OR pentrofane OR pertofran* OR pertofrin OR pertrofran OR petrofran* OR petylyl OR sertofren):ti,ab,kw) OR ('doxepin'/exp OR (adapin OR anten OR aponal OR curatin OR deptran OR desidox OR doneurin OR doxal OR doxepin* OR expan OR gilex OR mareen OR prudoxin OR quitaxon OR silenor OR sinequan OR sinquan OR sinquane OR zonalon):ti,ab,kw) OR ('imipramine'/exp OR (antidep OR antideprin OR apo-imipramine OR berkomin OR chrytemin OR daypress OR deprinol OR depsol OR depsonil OR ethipramine OR fronil OR imavate OR imidol OR imipramide OR imipramin* OR imiprin OR imizin OR imizine OR janimine OR melipramin* OR norpramine OR novopramine OR pramine OR presamine OR primonil OR pryleugan OR psychoforin OR psychoforine OR sermonil OR servipramine OR talpramin OR tofranil OR trofanil OR venefon):ti,ab,kw) OR ('nortriptyline'/exp OR (acetexa OR allegron OR altilev OR ateben OR atilev OR avantyl OR aventyl OR desitriptyline OR desmethylamitriptyline OR martimil OR noramitriptyline OR

noritren OR norline OR norpress OR nortrilen* OR nortriptylin* OR nortrix OR nortryptilin OR nortryptiline OR nortryptyline OR nortyline OR norventyl OR ortrip OR pamelor OR paxtibi OR psychostyl OR sensaval OR sensival OR vividyl):ti,ab,kw) OR ('protriptyline'/exp OR (amimetilina OR concordin OR maximed OR protriphyline OR protriptyline OR protryptiline OR protryptyline OR triptil OR vivactil):ti,ab,kw) OR ('trimipramine'/exp OR (apo-trimip OR herphonal OR rhotrimine OR sapilent OR stangyl OR sumontil OR surmontil OR trimepramine OR trimeprimin* OR trimepropimine OR trimipramine OR trimoprimine OR tripress OR tydamine):ti,ab,kw) OR ('tetracyclic antidepressant agent'/exp OR (tetracyclic NEAR/3 antidepress*):ti,ab,kw) OR ('beloxepin'/exp OR Beloxepin:ti,ab,kw) OR ('levoprotiline'/exp OR (levoprotiline OR levo-oxaprotiline OR levoxaprotiline):ti,ab,kw) OR ('maprotiline'/exp OR (kanopan OR ludiomil OR maprostad OR maprotilene OR maprotilin* OR melodil OR mirpan OR psymion OR retinyl OR urdiomil):ti,ab,kw) OR ('mianserin'/exp OR (athymil OR bolvidon OR investig OR lantanon OR lanthanon OR lerivon OR mianserin* OR miaxan OR norval OR serelan OR tetramide OR tolvan OR tolvin OR tolvon):ti,ab,kw) OR ('mirtazapine'/exp OR (6-azamianserin OR afloyan OR avanza OR calixta OR depreram OR esprital OR mepirzapin OR mirap OR mirataz OR mirta-tad OR mirtabene OR mirtadepi OR mirtalich OR mirtamor OR mirtapil OR mirtastad OR mirtazapin* OR mirtel OR mirtin OR mirtor OR mirzaten OR mitabor OR mizapin OR norset OR pharmataz OR promyrtil OR psidep OR remergil OR remergon OR remeron OR remirta OR rexer OR saxib OR zismirt OR zispin):ti,ab,kw) OR ('oxaprotiline'/exp OR oxaprotilin*:ti,ab,kw) OR ('teciptiline'/exp OR (setiptiline OR teciptiline OR teciptiline):ti,ab,kw) OR ('n methyl dextro aspartic acid receptor blocking agent'/exp OR ((NMDA OR n-methyl-d-aspart* OR n-methyl-dextro-aspart*) NEAR/3 (antagonist* OR block*)):ti,ab,kw) OR (neudexta OR neurodex OR nuedexta OR (dextromethorphan NEAR/3 quinidine)):ti,ab,kw OR (esgamda OR eskelan OR esketamin* OR esketiv OR falkieri OR ketanest-s OR keyzilen OR s-ketamin* OR sinmelan OR spravato OR vesierra):ti,ab,kw OR (adaxor OR akatinol OR almerzac OR alzantin OR alzedem OR axura OR biomentin OR cognomem OR demenco OR ebix OR ebixa OR ebixza OR mantinex OR mantomed OR marixino OR maruxa OR maryzola OR memabix OR memamed OR memando OR memantine OR memary OR memaxa OR memigmin OR memini OR memixa OR memolan OR mirvedol OR morysa OR namenda OR nemdatine OR nemedan OR polmatine OR uxamax OR valios OR zalatine OR zenmem):ti,ab,kw OR (acrescent OR arimenda OR balaxur OR namzaric):ti,ab,kw OR (neudexta OR neurodex OR zenvia OR (dextromethorphan NEAR/3 quinidine)):ti,ab,kw)

AND

('glioblastoma'/exp OR (gliobastoma* OR glyoblastoma* OR ((anaplastic OR grade-iv OR grade-4) NEAR/2 astrocytoma) OR (malignant NEAR/2 glioma)):ti,ab,kw)

NOT

('animal'/exp NOT 'human'/exp)

**Data management:**

Articles selected will be stored in Covidence for screening of studies and data extraction.

Selection process:

Two independent reviewers will assess remaining articles for relevance first based on titles and abstracts, and then will assess full-text articles for eligibility. Disagreements between reviewers will be resolved in both phases by either consensus or by a third reviewer.

**Data Collection Process:**

Each selected study will be distributed to two individuals for data extraction in duplicate using Covidence with preselected variables (see data items below). We anticipate no effort needed to contact authors of selected studies to obtain patient level data.

**Data items for extraction:**

- Study: (First author name followed by et al.)
- Year of publication
- Overall survival
- Effect size for antidepressant usage
- Odds ratio for antidepressant usage
- Hazard ratio for antidepressant usage
- Upper limit CI for each pre-defined outcome variable
- Lower limit CI for each pre-defined outcome variable
- Difference in survival
- Study size (number of patients in each treatment group)
- Standard Error (calculated)
- Demographic and patient enrollment characteristics

**Metadata:**

- Journal name where study was published.
- Year of publication
- Enrollment criteria

**Analysis approach:**

- Intention-to-treat vs per-protocol.
- Adherence to CONSORT or STROBE
- Potential sources of bias.

**Quality assessment/ Risk of bias analysis:**

Risk of bias will be determined for each study via the ROBINS-I tool. Quality will also be addressed by assessing compliance to research reporting guidelines such as STROBE. Competing interests in each study will be noted if any author had ties to industry particularly those funded by an industry sponsor.

**Strategy of data synthesis:**

We expect variability in patient selection among the studies. Therefore, we plan on using a random‐effects model with restricted maximum-likelihood estimation to perform. We plan on using an inconsistency index (I2) to assess for heterogeneity. We will also be calculating the mean difference in survival between treatment groups.

**Subgroup analysis:**

Separations based on antidepressant class and timing of therapy initiation, if available, will be made.

**Sensitivity analysis:** We will perform a sensitivity analysis by exploring how global effect sizes and p-values were affected by adjusting to the between-study variance parameter τ2. Statistical heterogeneity and the magnitude of heterogeneity will be assessed using Cochran χ2 tests and the I2 statistic, respectively. Publication bias will be assessed using the Egger test and visually using funnel plots. All statistical analyses were performed using R (version 4.3.1).^12^ Packages utilized will include the meta package.^13^ Alpha will be set at 0.05 and all test of significance will be 2-sided. Data and syntax used for the analysis will be made publicly available through GitHub.

**eAppendix 2**. Detailed search strategy employed for each database.

**Search Strategy:**

Concept 1 (Antidepressant Agents) AND Concept 2 (Glioblastoma) NOT ('animal'/exp NOT 'human'/exp)

(('antidepressant agent'/exp OR (anti-depress* OR antidepress* OR neurothymoleptic OR psychoenergizer OR thymoleptic OR thymolytic-agent):ti,ab,kw) OR ('serotonin uptake inhibitor'/de OR 'serotonin noradrenalin reuptake inhibitor'/de OR 'monoamine oxidase inhibitor'/exp OR (SSRI* OR SNRI* OR SSNRI* OR ((serotonin OR 'dual uptake' OR 'dual reuptake' OR noradrenalin OR monoamine OR dopamine OR norepinephrine OR MOA) NEAR/2 (inhibit*))):ti,ab,kw) OR ('citalopram'/de OR (acelopam OR adeprenal OR apo-cital OR aurex OR ceform OR celexa OR cilopress OR cinavol OR ciprager OR cipram OR cipramil OR cipraned OR ciprotan OR ciral OR citabax OR citacip OR citagen OR cital* OR citalon* OR citalopram OR citalostad OR citalox OR citalvir OR citapram OR citaxin OR citesint OR citopam OR citrol OR citronil OR cytalopram OR dalsan OR elopram OR exenadil OR frimaind OR futuril OR galopran OR humorap OR kaidor OR kitapram OR linisan OR lopracil OR lopraxer OR loxopram OR lupram OR malicon OR nitalapram OR oropram OR percitale OR pralotam OR pramital OR prefucet OR pricital OR prisdal OR psiconor OR renevil OR ricap OR ropramin OR selon OR sepram OR seralgan OR seregra OR serital OR seropram OR seror OR sintopram OR sotovon OR talam OR talosin OR varom OR vesema OR xadorek OR zanipram OR zeclicid OR zentius OR zitolex OR zyloram):ti,ab,kw) OR ('escitalopram'/de OR (aciprex OR amasci OR anxila OR apoescitaxin OR betesda OR cipralex OR citafort OR citram OR clomentin OR depralin OR depresinal OR diprex OR ecytara OR elicea OR entact OR escidivule OR escipram OR esciprex OR escirdec OR escital* OR escitalopram OR escitalpro OR escitil OR esertia OR eslorex OR esoprex OR espoza OR estan OR etalopro OR giachela OR heipram OR itakem OR lenuxin OR lexapro OR mersinol OR miraklide OR mozarin OR nexpram OR oroes OR pralex OR pramatis OR prameffex OR pramulex OR premalex OR prilect OR raldon OR ratice OR scippa OR serodeps OR seroplex OR serpentil OR servenon OR sipralexa OR symescital OR tepram OR zecidec OR zocital):ti,ab,kw) OR ('fluoxetine'/de OR (Actan OR adofen OR afeksin OR andep* OR animex-on OR ansilan OR auroken OR auscap OR bioxetin OR captaton OR daforin OR dagrilan OR depren OR deprex-leciva OR deprexetin OR deprexin OR deprizac OR deproxin OR diesan OR digassim OR elizac OR eufor OR exostrept OR felicium OR fldiss OR flotinal OR floxet OR fluctin* OR fludac OR flufran OR fluketin OR flunil OR flunirin OR fluocim OR fluohexal OR fluoksetin OR fluoksetyna OR fluox OR fluoxac OR fluoxeren OR fluoxetin* OR fluoxifar OR fluoxil OR fluoxin OR fluoxone OR fluoxtab OR fluronin OR flusac OR flustad OR flutin OR flutine OR fluxemed OR fluxen OR fluxet* OR fluxil OR fluxomed OR fluzac OR fluzak OR fokeston OR fontex OR foxetin OR foxtin OR fropine OR fuloren OR gerozac OR ladose OR lanclic OR lorien OR lovan OR luramon OR magrilan OR margrilan OR meropan OR modipran OR mutan OR nopres OR nuzak OR olena OR oxactin OR oxedep OR plazeron OR plinzene OR pragmaten OR prizma OR proctin OR prodep OR prosac OR prozac OR prozamel OR prozamin OR prozep OR prozit OR psipax OR psiquial OR qualisac OR rapiflux OR reconcile OR reneuron OR rowexetina OR salipax OR sanzur OR sarafem OR sartuzin OR selfemra OR serelsa OR seromex OR seronil OR sinzac OR sofelin OR tuneluz OR veritina OR xeredien OR zactin OR zepax OR zinovat):ti,ab,kw) OR ('paroxetine'/de OR

(arketis OR aropax OR aroxat OR brisdelle OR daparox OR deroxat OR dexorat OR divarius OR dropax* OR euplix OR eutimil OR frosinor OR mesafem OR motivan OR optipar OR paluxetil OR paluxon OR paroc OR parogen OR paroxedura OR paroxet* OR paxan OR paxil OR paxtine OR paxxet OR pexeva OR serestill OR sereupin OR seroxat OR setine OR syntopar OR tagonis OR paroxetine):ti,ab,kw) OR ('sertraline'/de OR (adjuvin OR ainim OR altisben OR altruline OR aremis OR asentra OR aserin OR asertin OR atruline OR besitran OR certorun OR contulen OR depreger OR dominum OR doxime OR enidap OR enore OR epilyd OR ferbrain OR fridep OR gladem OR lesefer OR lustral OR luxeta OR miravil OR neurosedine OR nudep OR sastium OR seltra OR semonic OR sercerin OR serimel OR serlain OR serlan OR serlift OR serlin OR serolux OR seromeg OR sertabal OR sertadepi OR sertagen OR sertral OR sertralet OR sertraline OR sertralon OR sertranat OR sertranex OR sertranorm OR sertranquil OR sertrone OR serunato OR setaloft OR sonalia OR sosser OR stimuloton OR tatig OR tresleen OR zolof OR zoloft OR zolotrin OR zortal OR zosert OR zotral):ti,ab,kw) OR ('desvenlafaxine'/exp OR (desvenlafaxine OR desmethylvenlafaxine OR ellefore OR khedezla OR norvenlafaxine OR Pristiq OR pristiqs):ti,ab,kw) OR ('duloxetine'/exp OR (ariclaim OR cymbalta OR drizalma OR dulane OR duloxetine OR duzela OR irenka OR nodetrip OR xeristar OR yentreve):ti,ab,kw) OR ('milnacipran'/exp OR (dalcipran OR fetzima OR impulsor OR ixel OR joncia OR levomilnacipran OR midalcipran OR milnacipran OR milnaneurax OR savella OR toledomin):ti,ab,kw) OR ('venlafaxine'/exp OR (alventa OR amphero OR apclaven OR 'arafaxina retard' OR axyven OR bonilux OR depefex OR deprevix OR dobupal OR duofaxin OR efaxine OR efectin OR efexor OR effexor OR effexstad OR elafax OR elify OR faxigen OR faxiprol OR faxiven OR faxolet OR fobiless OR genexin OR hapixed OR ireven OR lafactin OR lanvexin OR majoven OR olwexya OR oriven OR pracet OR prefaxine OR serosmine OR sigven OR sivion OR sunveniz OR symfaxin OR tonpular OR trevilor OR trewilor OR vandral OR vaxor OR vedixal OR velafax OR velaxin OR velept OR venaxx OR vencarm OR venex OR venla OR venlablue OR venlabrain OR venladex OR venlafab OR venlafaxin OR venlafaxina OR venlafaxine OR venlafex OR venlagamma OR venlalic OR venlaneo OR venlasov OR venlatev OR venlax OR venlaxin OR venlaxor OR venlazid OR venlectine OR venlofex OR venlor OR venprimeven OR vensir OR vensuerteven OR venxin OR venzip OR vexarin OR viepax OR xenalven OR zacalen OR zaredrop OR zarelis OR zarelix OR venlafaxine):ti,ab,kw) OR ('atypical antidepressant agent'/exp) OR ('amfebutamone'/exp OR (amfebutamone OR aplenzin OR budeprion OR buprion-hydrochloride OR bupropin OR bupropion OR buproprion OR buxon OR elontril OR forfivo OR odranal OR quomem OR quomen OR wellbatrin OR wellbutrin OR zyban):ti,ab,kw) OR ('mirtazapine'/exp OR (azamianserin OR afloyan OR avanza OR calixta OR depreram OR esprital OR mepirzapin OR mirap OR mirataz OR mirtabene OR mirtadepi OR mirtalich OR mirtamor OR mirtapil OR mirtastad OR mirtazapin* OR mirtel OR mirtin OR mirtor OR mirzaten OR mitabor OR mizapin OR norset OR pharmataz OR promyrtil OR psidep OR remergil OR remergon OR remeron OR remirta OR rexer OR saxib OR yarocen OR zismirt OR zispin):ti,ab,kw) OR ('nefazodone'/exp OR (dutonin OR nefadar OR nefazodone OR reseril OR serzone OR serzonil):ti,ab,kw) OR ('trazodone'/exp OR (azonz OR beneficat OR bimaran OR deprax OR depresil OR depyrel OR desirel OR desyrel OR manegan OR molipaxin OR oleptro OR pesyrel OR pragazone OR pragmarel OR pragmazone OR reslin OR taxagon OR thombran OR thromban OR thrombran OR tombran OR tradozone OR trasodon OR trasodone OR trazodil OR trazodon OR trazodone OR trazolan OR trialodine

OR trittico):ti,ab,kw) OR ('vilazodone'/exp OR (viibryd OR vilazodone):ti,ab,kw) OR ('vortioxetine'/exp OR (brintellix OR trintellix OR vortioxetine):ti,ab,kw) OR ('isocarboxazid'/exp OR (bmih OR enerzer OR isocarboazide OR isocarboxacid OR isocarboxazid* OR marplan):ti,ab,kw) OR ('phenelzine'/exp OR (fenelzine OR fenizin OR mao-rem OR nardelzine OR nardil OR phenalzine OR phenelzin* OR phenethylhydrazine OR phenylethylhydrazine OR stinerval):ti,ab,kw) OR ('selegiline'/exp OR (anipryl OR antiparkin OR apo-selegiline OR atapryl OR deprenaline OR deprenil OR deprenyl OR egibren OR eldeprine OR eldepryl OR elegelin OR emsam OR julab OR julegil OR jumex OR jumexal OR kinline OR l-deprenyl OR levo-deprenyl OR mao-b OR maotil OR movergan OR niar OR otrasel OR parkryl OR phenylisopropylmethylpropynylamine OR plurimen OR procythol OR sedicel OR sefmex OR seledat OR selegil OR selegiline OR selegos OR selgene OR selgin OR xilopar OR zelapar OR selegiline):ti,ab,kw) OR ('tranylcypromine'/exp OR (jatrosom OR parmodalin OR parnate OR parnitene OR parnitine OR trancilpromine OR trancylpromine OR trancylprominesulfate OR trancylprominesulphate OR tranilacipromina OR transamine OR tranylcypomia OR tranylcypromide OR tranylcypromin* OR tylciprine):ti,ab,kw) OR ('tricyclic antidepressant agent'/exp OR (tricyclic NEAR/3 antidepress*):ti,ab,kw) OR ('amitriptyline'/exp OR (adepress OR adepril OR ambivalon OR amilit OR amineurin OR amiplin OR amiprin OR amirol OR amitid OR amitril OR amitrip OR amitriptylene OR amitriptylin* OR amitriptylinumhydrochloride OR amitryptiline OR amitryptilline OR amitryptine OR amitriptyline OR amyline OR amytril OR amytriptiline OR amytriptyline OR amytryptiline OR amyzol OR anapsique OR antalin OR antitriptyline OR astilin OR damilen* OR damitriptyline OR damylene OR deprelio OR domical OR elatrol* OR elavil OR enafon OR endep OR enovil OR etafon OR etafron OR euplit OR lantron OR laroxal OR laroxyl OR lentizol OR miketorin OR novoprotect OR ormal OR pinsaun OR proheptadien OR qualitriptine OR redomex OR sarboten-retard OR sarotard OR saroten* OR sarotex OR stelminal OR sylvemid OR syneudon OR syneydon OR teperin OR terepin OR trepiline OR tridep OR tripta OR triptanol OR triptizol OR triptyl OR triptyline OR trynol OR tryptanol OR tryptizol OR trytomer OR uxen OR vanatrip):ti,ab,kw) OR ('amoxapine'/exp OR (adisen OR amoxan OR amoxapin* OR asendin OR asendis OR defanyl OR demolox OR moxadil):ti,ab,kw) OR ('desipramine'/exp OR (demethylimipramine OR deprexan OR desimipramine OR desipramin OR despiramine OR desipramine OR desmethyl-imipramin* OR desmethylimipramin* OR nebril OR norimipramine OR norpramin* OR nortimil OR pentrofane OR pertofran* OR pertofrin OR pertrofran OR petrofran* OR petylyl OR sertofren):ti,ab,kw) OR ('doxepin'/exp OR (adapin OR anten OR aponal OR curatin OR deptran OR desidox OR doneurin OR doxal OR doxepin* OR expan OR gilex OR mareen OR prudoxin OR quitaxon OR silenor OR sinequan OR sinquan OR sinquane OR zonalon):ti,ab,kw) OR ('imipramine'/exp OR (antidep OR antideprin OR apo-imipramine OR berkomin OR chrytemin OR daypress OR deprinol OR depsol OR depsonil OR ethipramine OR fronil OR imavate OR imidol OR imipramide OR imipramin* OR imiprin OR imizin OR imizine OR janimine OR melipramin* OR norpramine OR novopramine OR pramine OR presamine OR primonil OR pryleugan OR psychoforin OR psychoforine OR sermonil OR servipramine OR talpramin OR tofranil OR trofanil OR venefon):ti,ab,kw) OR ('nortriptyline'/exp OR (acetexa OR allegron OR altilev OR ateben OR atilev OR avantyl OR aventyl OR desitriptyline OR desmethylamitriptyline OR martimil OR noramitriptyline OR

noritren OR norline OR norpress OR nortrilen* OR nortriptylin* OR nortrix OR nortryptilin OR nortryptiline OR nortryptyline OR nortyline OR norventyl OR ortrip OR pamelor OR paxtibi OR psychostyl OR sensaval OR sensival OR vividyl):ti,ab,kw) OR ('protriptyline'/exp OR (amimetilina OR concordin OR maximed OR protriphyline OR protriptyline OR protryptiline OR protryptyline OR triptil OR vivactil):ti,ab,kw) OR ('trimipramine'/exp OR (apo-trimip OR herphonal OR rhotrimine OR sapilent OR stangyl OR sumontil OR surmontil OR trimepramine OR trimeprimin* OR trimepropimine OR trimipramine OR trimoprimine OR tripress OR tydamine):ti,ab,kw) OR ('tetracyclic antidepressant agent'/exp OR (tetracyclic NEAR/3 antidepress*):ti,ab,kw) OR ('beloxepin'/exp OR Beloxepin:ti,ab,kw) OR ('levoprotiline'/exp OR (levoprotiline OR levo-oxaprotiline OR levoxaprotiline):ti,ab,kw) OR ('maprotiline'/exp OR (kanopan OR ludiomil OR maprostad OR maprotilene OR maprotilin* OR melodil OR mirpan OR psymion OR retinyl OR urdiomil):ti,ab,kw) OR ('mianserin'/exp OR (athymil OR bolvidon OR investig OR lantanon OR lanthanon OR lerivon OR mianserin* OR miaxan OR norval OR serelan OR tetramide OR tolvan OR tolvin OR tolvon):ti,ab,kw) OR ('mirtazapine'/exp OR (6-azamianserin OR afloyan OR avanza OR calixta OR depreram OR esprital OR mepirzapin OR mirap OR mirataz OR mirta-tad OR mirtabene OR mirtadepi OR mirtalich OR mirtamor OR mirtapil OR mirtastad OR mirtazapin* OR mirtel OR mirtin OR mirtor OR mirzaten OR mitabor OR mizapin OR norset OR pharmataz OR promyrtil OR psidep OR remergil OR remergon OR remeron OR remirta OR rexer OR saxib OR zismirt OR zispin):ti,ab,kw) OR ('oxaprotiline'/exp OR oxaprotilin*:ti,ab,kw) OR ('teciptiline'/exp OR (setiptiline OR teciptiline OR teciptiline):ti,ab,kw) OR ('n methyl dextro aspartic acid receptor blocking agent'/exp OR ((NMDA OR n-methyl-d-aspart* OR n-methyl-dextro-aspart*) NEAR/3 (antagonist* OR block*)):ti,ab,kw) OR (neudexta OR neurodex OR nuedexta OR (dextromethorphan NEAR/3 quinidine)):ti,ab,kw OR (esgamda OR eskelan OR esketamin* OR esketiv OR falkieri OR ketanest-s OR keyzilen OR s-ketamin* OR sinmelan OR spravato OR vesierra):ti,ab,kw OR (adaxor OR akatinol OR almerzac OR alzantin OR alzedem OR axura OR biomentin OR cognomem OR demenco OR ebix OR ebixa OR ebixza OR mantinex OR mantomed OR marixino OR maruxa OR maryzola OR memabix OR memamed OR memando OR memantine OR memary OR memaxa OR memigmin OR memini OR memixa OR memolan OR mirvedol OR morysa OR namenda OR nemdatine OR nemedan OR polmatine OR uxamax OR valios OR zalatine OR zenmem):ti,ab,kw OR (acrescent OR arimenda OR balaxur OR namzaric):ti,ab,kw OR (neudexta OR neurodex OR zenvia OR (dextromethorphan NEAR/3 quinidine)):ti,ab,kw)

AND

('glioblastoma'/exp OR (gliobastoma* OR glyoblastoma* OR ((anaplastic OR grade-iv OR grade-4) NEAR/2 astrocytoma) OR (malignant NEAR/2 glioma)):ti,ab,kw)

NOT

('animal'/exp NOT 'human'/exp)

**eAppendix 3**. Results of sensitivity analysis conducted via leave-one-out analysis


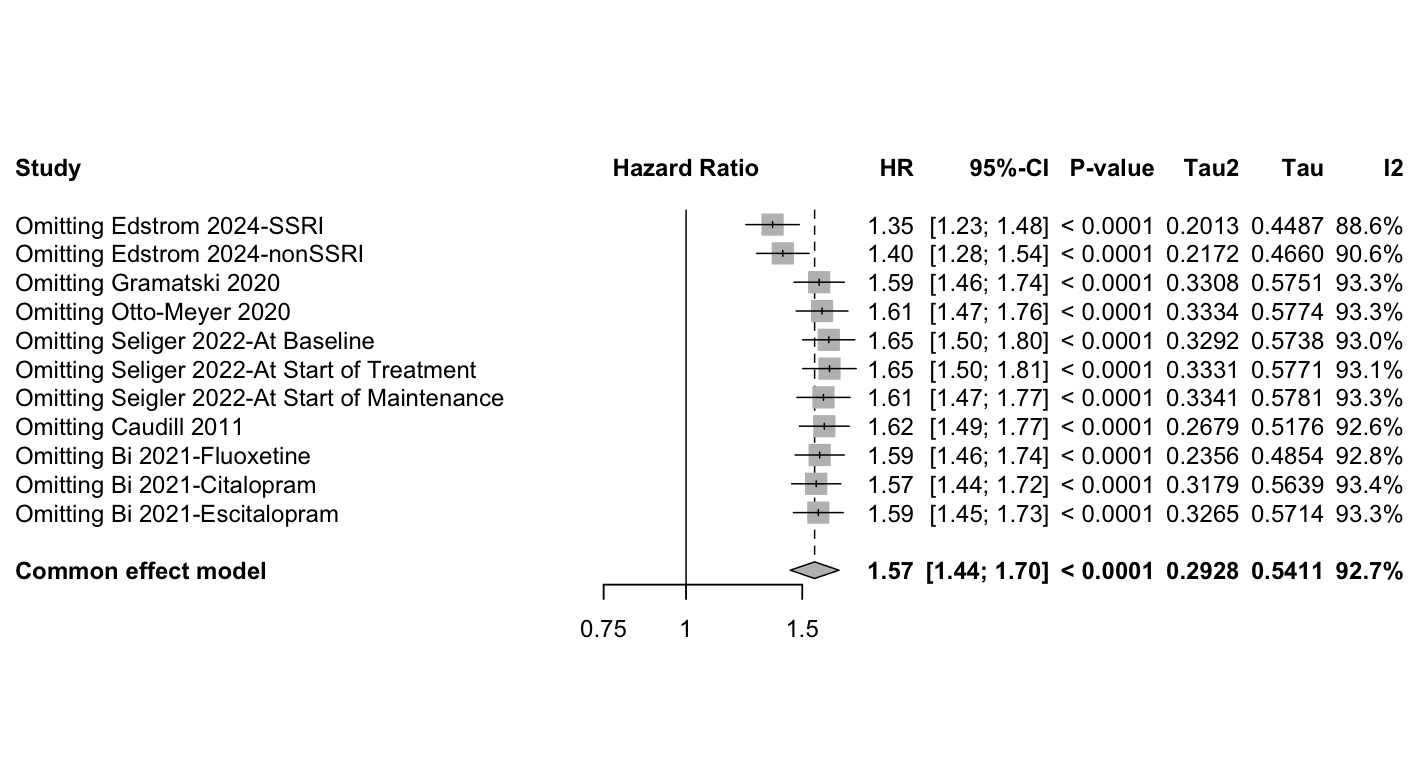


**eAppendix 4**. Copas-Like Selection Model Sensitivity Analysis

**
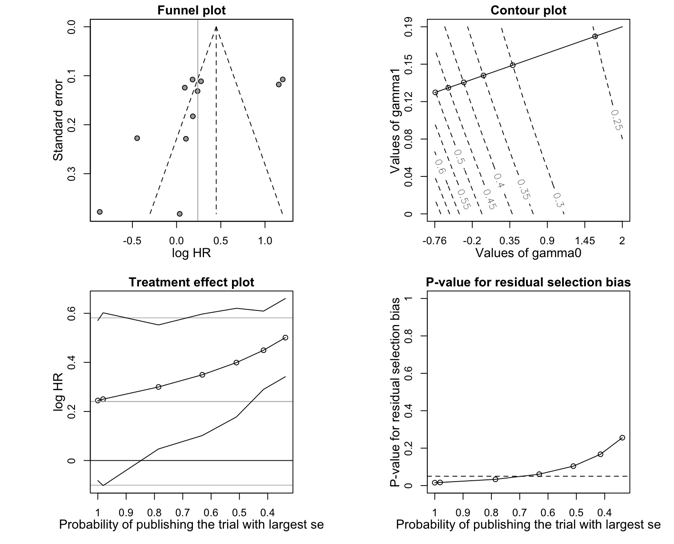
**

Results of Sensitivity Analysis via Copas-like selection model

| **Percent Published** | **HR** | **95%-CI** | τ^2^ | τ | **p-value adjusted** | **Probability of Publication** | **N** |
| --- | --- | --- | --- | --- | --- | --- | --- |
| 1 | 1.277 | [0.9214; 1.7697] | 0.2614 | 0.5113 | 0.142 | 0.0154 | 0 |
| 0.9812 | 1.2841 | [0.9029; 1.8261] | 0.2569 | 0.5069 | 0.1641 | 0.0166 | 0 |
| 0.7857 | 1.3497 | [1.0482; 1.7379] | 0.2281 | 0.4776 | **0.0201** | 0.0334 | 1 |
| 0.6311 | 1.4183 | [1.1072; 1.8169] | 0.2083 | 0.4564 | **0.0057** | 0.0609 | 3 |
| 0.5106 | 1.4905 | [1.1944; 1.8599] | 0.1933 | 0.4397 | **0.0004** | 0.1038 | 5 |
| 0.4148 | 1.5676 | [1.3365; 1.8386] | 0.1816 | 0.4262 | **< 0.0001** | 0.1674 | 8 |
| 0.3376 | 1.6504 | [1.4072; 1.9358] | 0.1726 | 0.4154 | **< 0.0001** | 0.2559 | 11 |
| Adjusted estimate: | 1.4183 | [1.1072;1.8169] | 0.2083 | 0.4564 | 0.0057 | 0.0609 | 3 |
| Unadjusted estimate: | 1.2718 | [0.9043;1.7888] | 0.2928 | 0.5411 | 0.1671 | 0.1671 |  |

**eAppendix 5**. Funnel Plot for Egger’s Test. Egger’s test did not indicate significant evidence of publication bias (p=0.122)


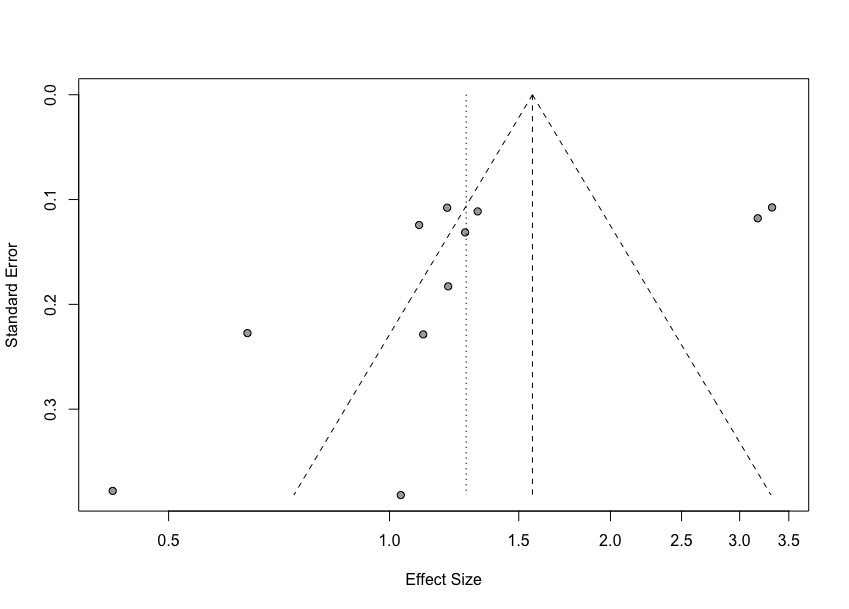


**eAppendix 6.** Risk of Bias assessment using the ROBINS-I tool

|  | Risk of Bias Domains | | |  |  |  |  |  |
| --- | --- | --- | --- | --- | --- | --- | --- | --- |
| Study | Bias Due to Confounding | Bias in Selection of Participants | Bias in Classification of Interventions | Bias Due to Deviations from Intended Interventions | Bias Due to Missing Data | Bias in Measurement of Outcomes | Bias in Selection of Reported Result | Overall Bias |
| Edstrom 2024 | Moderate | Moderate | Moderate | Moderate | Low | Low | Moderate | Moderate |
| Gramatzki 2020 | Moderate | Low | Low | Moderate | Low | Low | Moderate | Moderate |
| Otto-Meyer 2020 | Serious* | Moderate | Low | Low | Low | Low | Moderate | Serious |
| Seliger 2022 | Moderate | Moderate | Serious | Low | Low | Low | Low | Serious |
| Caudill 2011 | Serious* | Moderate | Moderate | Low | Low | Low | Low | Serious |
| Bi 2021 | Serious* | Low | Moderate | Low | Low | Low | Low | Serious |

**eAppendix 7**. Grading of Recommendations Assessment, Development, and Evaluation (GRADE) assessment for quality of evidence.

| **Certainty assessment** | | | | | | | **№ of patients** | | **Effect** | | **Certainty** | **Importance** |
| --- | --- | --- | --- | --- | --- | --- | --- | --- | --- | --- | --- | --- |
| **№ of studies** | **Study design** | **Risk of bias** | **Inconsistency** | **Indirectness** | **Imprecision** | **Other considerations** | **Antidepressant** | **No Antidepressants** | **Relative (95% CI)** | **Absolute (95% CI)** |  |  |
| **Overall Survival** | | | | | | | | | | | | |
| 6 | Observational Studies | Not Serious | Serious | not serious | Serious | none | 1093 | 7176 | - | odds ratio **0 1.27**  (0.90 higher to 1.97 higher) | ⨁⨁∅∅ Low |  |

**eAppendix 8.** Adherence to Reporting Guidelines

| Adherence to Strobe | Items met |
| --- | --- |
| Edstrom 2024 | 21/22 |
| Gramatzki 2020 | 22/22 |
| Otto-Meyer 2020 | 20/22 |
| Seliger 2022 | 22/22 |
| Caudill 2011 | 21/22 |
| Bi 2021 | 20/22 |
